# Correlation of heat shock protein 1 expression with progression and prognosis of non-small cell lung cancer

**DOI:** 10.3389/fonc.2025.1553248

**Published:** 2025-05-06

**Authors:** Rui Wang, Rongqi Guo, Tianyi Huang, Yu Lu, Weisong Zhang, Yihao Wang, Qinggan Ni, Jianxiang Song, Xia Li

**Affiliations:** ^1^ Department of Thoracic Surgery, Affiliated Hospital 6 of Nantong University, Yancheng Third People’s Hospital, Yancheng, Jiangsu, China; ^2^ Medical College of Nantong University, Nantong, Jiangsu, China; ^3^ Department of Burns and Plastic Surgery, Yancheng Clinical College of Xuzhou Medical University, The First People’s Hospital of Yancheng, Yancheng, Jiangsu, China; ^4^ Department of General Medicine, Affiliated Hospital 6 of Nantong University, Yancheng Third People’s Hospital, Yancheng, Jiangsu, China

**Keywords:** HSPH1, NSCLC, biomarker, bioinformatics analysis, prognosis, progression

## Abstract

**Aims:**

To investigate the expression and clinical significance of heat shock protein H1 (HSPH1) in non-small cell lung cancer (NSCLC). To provide new and reliable biomarkers for the treatment of NSCLC.

**Main methods:**

Various public databases were used to analyse the molecular characteristics, disease relevance and expression differences of HSPH1 and to investigate the correlation between HSPH1 expression and prognosis in NSCLC patients. MsigDB and Molecular signatures database were used for enrichment analysis. The HSPH1 protein interaction network was constructed using the STRING database. In addition, immune infiltration analysis was performed. Subsequently, the expression levels of HSPH1 in Lung cancer cell lines, human NSCLC tissues and normal tissues were determined using various experiments. Finally, a retrospective analysis was performed to determine whether the expression level of HSPH1 was associated with the clinical characteristics and prognosis of NSCLC patients.

**Results:**

The high expression of HSPH1 in NSCLC was significantly correlated with the clinical characteristics and poor prognosis of patients, and it may affect the progression of NSCLC by forming a regulatory network with molecules such as HSPA8, BAG2, etc., and its mechanism may involve the regulation of the P53 signalling pathway, G2M_CHECKPOINT and mTORC1_SGNALING. Experiments confirmed that HSPH1 was also highly expressed in NSCLC tissues and cells.

**Conclusion:**

HSPH1 can be used as a potential diagnostic and prognostic marker for NSCLC, and its involvement in NSCLC progression and immune regulation may be one of its therapeutic targets.

## 1 Introduction

Lung cancer is the leading cause of cancer-related death worldwide (1). Non-small cell lung cancer (NSCLC) accounts for more than 85% of all cases, and its five-year survival rate is less than 20%, mainly due to late diagnosis, metastasis tendency, and treatment resistance (2, 3). Although advances in targeted therapy and immunotherapy have significantly improved the prognosis of some patients, most patients still face limited efficacy or risk of recurrence due to tumour heterogeneity and molecular mechanism complexity (4). Therefore, exploring the key molecules and their regulatory networks driving the progress of NSCLC is of great significance for the development of novel biomarkers and precision treatment strategies.

Heat shock proteins (HSPs) are a class of genetically highly conserved protein molecules that act as molecular chaperones, protecting proteins from destruction and helping to repair misfolded protein molecules (5). Heat shock protein H1 (HSPH1), also known as HSPH110 or HSP105, is a member of the HSP105/110 family of heat shock proteins (6). In addition to its protective function, HSPH1 is also associated with tumour cell proliferation, differentiation, invasion and metastasis (7). Some studies have reported that HSPH1 is highly expressed in a variety of cancers, including digestive tract tumours, head and neck tumours and haematological tumours, while it is less expressed in normal tissues, and that the expression of HSPH1 is significantly correlated with the clinical stage and prognosis of a variety of cancers (8–13). Notably, a study in lung cancer found that silencing HSPH1 significantly increased the anti-tumour effect of gefitinib in non-small cell lung cancer (6), suggesting that HSPH1 may be a potential biomarker for the diagnosis, targeted therapy and prognostic evaluation of a variety of human tumours. However, the expression of HSPH1 in NSCLC and its correlation with clinical features have not been reported.

In this study, we analysed the expression level of HSPH1 in NSCLC and its value for diagnosis and prognosis by bioinformatics methods, explored the potential mechanism of HSPH1 in NSCLC by pathway enrichment analysis and immune infiltration analysis, and further validated the bioinformatics results by experiments and collection of clinical samples, with the aim of providing new ideas for early diagnosis, targeted intervention and We hope to provide new ideas for early diagnosis, targeted intervention and prognostic evaluation of NSCLC.

## 2 Materials and methods

### 2.1 Patient population and tissue samples

A sample of 95 NSCLC patients admitted to the hospital of the Third People’s Hospital of Yancheng City (the Sixth Affiliated Hospital of Nantong University) between May 2015 and December 2019 was included in the study. In addition to clinical data (including age, gender, smoking index, clinical stage, and histology stage), we collected samples of the patient’s tumour and peritumour tissue. It is worth noting that well differentiation is defined as the tumour cell morphology is close to normal, and the acinar/squamous structure is intact. Poor differentiation is characterised by significant cell atypia and structural disturbance. Smoking index = number of daily cigarettes × number of years of smoking. All 7 pairs of tissues used for Western blot experiments were immediately placed in an ice box after surgical excision and stored in liquid nitrogen according to the principle of asepsis after timely sampling, and all tissues used for immunohistochemistry were immediately fixed in 10% formalin solution after surgical excision, then cut into 5 μm thick frozen sections and stored at -80 degrees Celsius. The inclusion criteria for patients were (1) NSCLC confirmed by pathological review of tissue. (2) Age between 18 and 90 years (3) All patients met the criteria for surgery and did not undergo neoadjuvant radiotherapy (4) No obvious contraindications to surgery (5) No obvious endocrine and metabolic diseases (6) No history of psychiatric disorders. All participants were followed up for more than 5 years. The study was approved by the Ethics Committee of the Third People’s Hospital of Yancheng City (Approval Number:LS20150623), informed consent was obtained from all participating patients and/or their legal guardians, and the study had to be conducted in strict accordance with the Declaration of Helsinki.

### 2.2 HSPH1 gene information

Human chromosomes and subcellular locations of HSPH1 were visualised using the GeneCards database (https://www.genecards.org/) (14).The OPENTARGET platform (https://platform.opentargets.org/) (15) identifies the gene HSPH1 in disease Role. Differential expression of the HSPH1 gene in pan-cancer visualised by the TIMER online database (http://timer.cistrome.org/) (16).

### 2.3 Data download

The TCGA database (https://portal.gdc.cancer.gov/), the largest repository of cancer gene information, contains data including gene expression data, copy number variations, single-nucleotide polymorphisms (SNPs), and other data. We downloaded the raw mRNA expression data, which comprised a total of 1153 samples. Among these, the normal group (n=110) and the tumour group (n=1043) were used for subsequent analysis.

### 2.4 Differential expression analysis

The expression levels of HSPH1 in NSCLC and the potential relationship with clinical staging were analysed through the UALCAN online website (https://ualcan.path.uab.edu/) (17).

The ENCORI online database (https://rnasysu.com/encori/) analyses the expression levels of HSPH1 in tumour tissues and normal tissues.

Differential expression of HSPH1 at the protein level was then investigated using the online database Human Protein Atlas (HPA, http://www.proteinatlas.org/). Immunohistochemical staining of clinical NSCLC tissues and normal lung epithelial tissues was performed using the HSPH1 specific antibody CAB002060.

Expression difference analyses were performed using the ANOVA algorithm in the Gene Expression Profiling Interactive Analysis 2 (GEPIA 2) online database (http://gepia2.cancer-pku.cn/#index).

### 2.5 Kaplan–Meier plot analysis

The Kaplan-Meier online database (https://kmplot.com/analysis/) (18) analysed HSPH1 gene expression in three different NSCLC microarray data (235573_at,208744_x_at,206976_s_at) based on risk ratio (HR) and log-rank p-value in relation to patients’ Overall Survival(OS) and First-progression Survival (FPS).

### 2.6 Correlation expression analysis

The STRING database (http://string-db.org) (19) was utilised to construct the HSPH1 protein-protein interaction (PPI) network. PPIs with an interaction score of >-0.40 were selected for visualisation.

### 2.7 Gene set enrichment analysis

The patients were divided into two groups based on the expression of their key genes, and the differences in signalling pathways between the two groups were subsequently analysed using GSEA. The background gene set was obtained from the MsigDB database (version 7.0) and comprised the annotated gene set of subtype pathways. This was used to conduct a differential expression analysis of pathways between different subgroups. The gene sets that were significantly enriched (adjusted p-value less than 0.05) were then ranked according to the concordance score. GSEA analysis is a commonly used method for exploring the close relationship between disease typing and biological significance (20).

### 2.8 Gene set variation analysis

Gene set variation analysis (GSVA) is a non-parametric and unsupervised method for assessing gene set enrichment in transcriptomes. GSVA transforms gene-level changes into pathway-level changes by integrally scoring gene sets of interest to determine the biological functions of the samples. In the present study, GSVA analysis used the Hallmark genome (50 pathways, source: MSigDB v7.5.1), focusing on screening pathways related to stress response (HALLMARK_HYPOXIA), DNA repair (HALLMARK_DNA_REPAIR) and immune regulation. Download link of genome set: https://www.gsea-msigdb.org/gsea/msigdb.

### 2.9 Immune infiltration analysis

The CIBERSORT method is a widely employed methodology for the assessment of immune cell types within the microenvironment. The method is founded upon the tenets of support vector regression and back-convolutional analysis of the expression matrix of immune cell subtypes. The method comprises 547 biomarkers that distinguish 22 human immune cell phenotypes, including T-cell, B-cell, plasma cell, and myeloid cell subpopulations. In this study, patient data were analysed using the CIBERSORT algorithm, which was employed to infer the relative proportions of the 22 immune-infiltrating cells and to correlate gene expression as well as immune cell content (21).

### 2.10 Cell culture

The cell lines utilised in this research were acquired from Wuhan Pricella Biotechnology, Inc., and comprised the following: A549, H1299, H1734, H838, HCC827, Beas-2b, and H1975. All cell cultures were maintained following the standardised protocol using Roswell Park Memorial Institute (RPMI) 1640 medium along with Dulbecco’s modified Eagle medium (DMEM). The foundational medium was a mixture of RPMI 1640 and DMEM, supplemented with 10% foetal bovine serum and 1% dual-antibiotic (penicillin + streptomycin) to formulate the complete medium. Cells were incubated in T25 cell culture flasks with perforations, each containing 5 ml of complete medium, and maintained in an incubator set at 37°C (with 5% CO_2_). Cells in the logarithmic growth phase, demonstrating optimal growth characteristics, were prepared for use in ensuing experiments. All materials mentioned above were sourced from Wuhan Punosai Biological Company in China.

### 2.11 Western blotting

Total proteins were extracted from samples of NSCLC, as well as surrounding tissue, and various cell lines using RIPA buffer (Beyotime, China). To quantify the protein concentrations, a BCA kit (Beyotime, China) was employed. The extracted protein samples underwent separation through a 12.5% SDS-PAGE gel (Epizyme, China), after which they were transferred to PVDF membranes (Millipore, USA) for further analysis. Following the transfer, the membranes were incubated in a 5% skimmed milk solution for a duration of two hours to block nonspecific binding sites. After this incubation period, the membranes were washed with TBST to remove excess blocking agent and were subsequently treated with primary antibodies, specifically anti-GAPDH (Proteintech, China) and anti-HSPH1 (Proteintech, China). The membranes remained in a chilled environment at 4°C overnight to allow for optimal antibody binding. After the overnight incubation, the membranes were subjected to three washes with TBST to ensure thorough removal of unbound antibodies. They were then incubated at room temperature, shielded from light, with a secondary antibody for one hour. Following this incubation, the membranes were washed again three times with TBST. The final step involved the development of the proteins on the membranes using an Enhanced Chemiluminescence Detection Kit (Ncmbio, China), which was performed under a Tanon-5200multl imaging system. The grey values of the developed bands were analysed using Image J software, utilising GAPDH as an internal reference for accurate measurement.

### 2.12 Immunohistochemistry

The specimens underwent fixation using 10% neutral formalin, followed by dehydration through a gradient series of ethanol, embedding in paraffin, and sectioning at a thickness of 7 μm. The expression of HSPH1 (rabbit polyclonal antibody, Proteintech, China) was determined using the immunohistochemical SP technique. The paraffin sections were subjected to deparaffinisation using xylene (China Pharmaceuticals) and then autoclaved for antigen retrieval. Following this, a 3% hydrogen peroxide solution (Sinopharm, China) was applied to inhibit endogenous peroxidase activity. To minimise non-specific binding of proteins, the sections were subsequently incubated with normal goat serum (Phygene, China) for 20 minutes. Drops of HSPH1 at a dilution of 1:100 were then left to incubate overnight at 4 °C. After a 15-minute incubation at room temperature with the secondary antibody, colour development was achieved using 3,3-diaminobenzidine (DAB) (Beyotime, China) for a duration of 3 to 5 minutes. The specimen was then stained with haematoxylin (Phygene, China), dehydrated, and mounted using neutral gum. Finally, observations were made with an Olympus BX83 fluorescence microscope (Japan). The results were assessed using the following approach: Blind examination of HSPH1 staining in tumour and non-tumour tissues by two pathologists. Three visual fields were selected to examine the proportion of positive cells and the intensity of cell staining. Immunohistochemical staining was evaluated according to staining intensity and proportion of positive cells, based on an immunoreactivity score (IRS). Strength scores were as follows: 0 (negative), 1 (weakly positive), 2 (moderately positive), and 3 (strongly positive). Quantitative scores were recorded according to the proportion of HSPH1 positive cells in four categories (1 (0% - 25%), 2 (26% - 50%), 3 (51% - 75%), and 4 (76% - 100%). The IRS (product of intensity score and number score) range from 0 to 12: IRS 0–3 and 4–12 represent low and high HSPH1 expression, respectively.

### 2.13 Immunofluorescent staining

Round coverslips were placed in 6-well culture plates, followed by seeding the cell suspension and culture in complete medium for 48–72 hours. After removing the medium, cells were briefly rinsed three times with phosphate-buffered saline (PBS) and fixed with 4% paraformaldehyde (PFA) at room temperature for 30 minutes. Fixed cells were then thoroughly washed with pre-cooled PBS (4°C) three times (5 minutes per wash).To enhance membrane permeability, samples were permeabilised with PBS containing 0.1-0.25% Triton X-100 for 10 minutes. Non-specific binding sites were blocked with 10% goat serum (Beyotime, China) at 37°C for 30 minutes. Primary antibodies were applied and incubated overnight at 4°C. The next day, samples were washed extensively with PBS (3 × 5 minutes) and incubated with TRITC-conjugated goat anti-rabbit IgG secondary antibodies (Beyotime, China) at room temperature for 1 hour, followed by additional PBS washes. Nuclear counterstaining was performed using DAPI solution (5 μg/mL) under dark conditions at room temperature for 10 minutes. Finally, coverslips were mounted with anti-fade mounting medium (Phygene, China) to preserve fluorescence signals. Fluorescence imaging was conducted using an Olympus BX83 fluorescence microscope (Japan), with consistent acquisition parameters across experimental groups.

### 2.14 Statistical analysis

The data were analysed using the statistical software package SPSS 25.0 (Chicago, IL, USA). The independence of categorical variables was tested using the chi-square test. Furthermore, Kaplan-Meier analyses were conducted to evaluate differences in survival. Variables with a p-value of less than 0.1 in the univariate analysis were included in the Cox proportional hazards model for multivariate analysis. Statistical significance was determined by a P-value of less than 0.05. In addition, we performed univariate and multivariate Cox proportional risk regression analyses using information on NSCLC samples downloaded from the TCGA database, and use the “forestplot” package to create a forest plot for a visual display of the P-value, hazard ratio (HR), and 95% confidence interval (CI) for each variable.

## 3 Results

### 3.1 HSPH1 localisation, expression, related diseases and pan-cancer analysis

**Figure 1:**
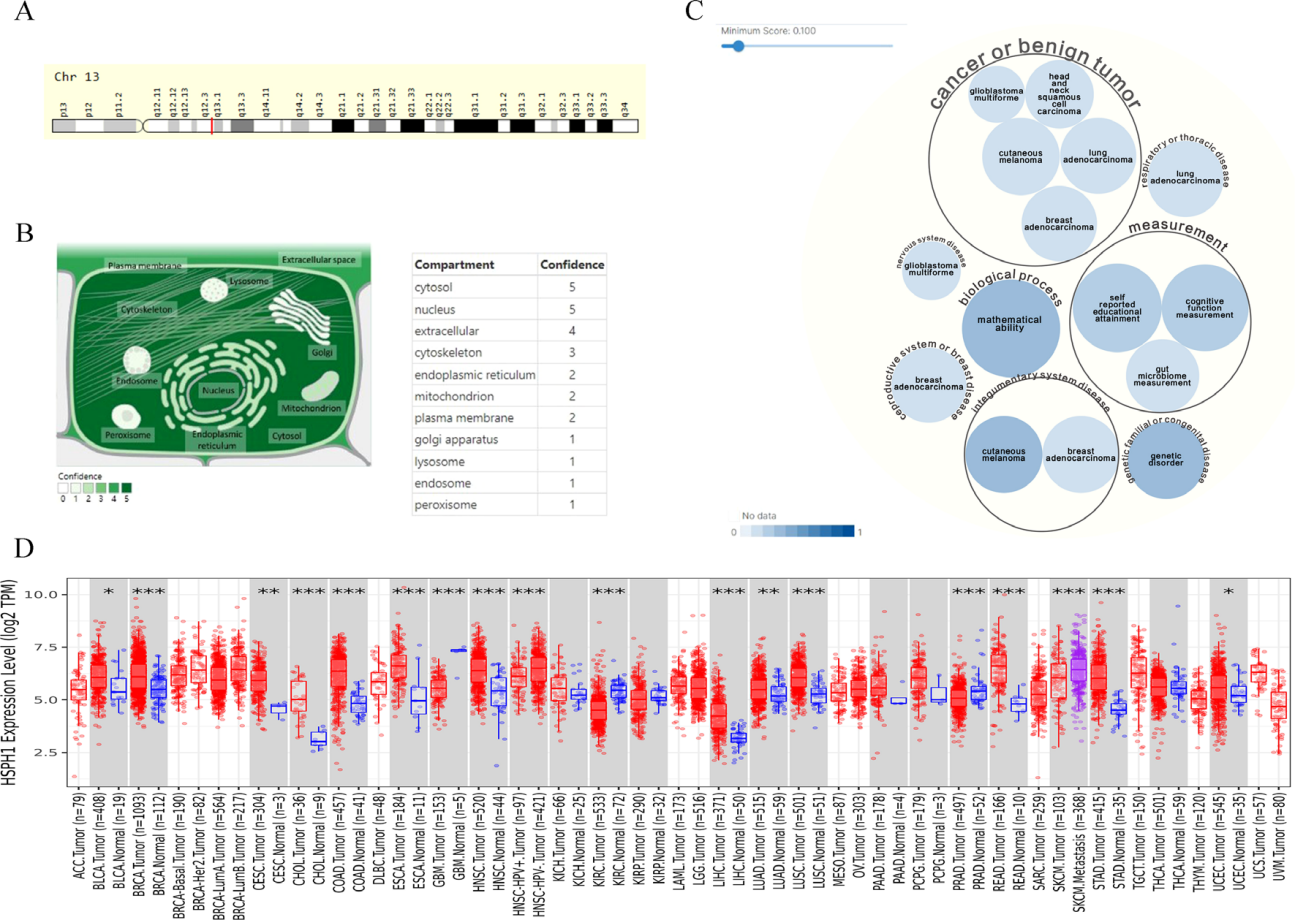
HSPH1 gene characterisation and disease correlation. **(A)** Chromosomal localisation of the HSPH1 gene. **(B)** Subcellular localisation of the protein encoded by the HSPH1 gene. **(C)** HSPH1 gene-disease correlation network. **(D)** TIMER database showing differential expression of HSPH1 in pan-cancer tissues. (*P < 0.05, **P < 0.01, ***P < 0.001).

HSPH1 is located on human chromosome 13 (**Figure 1A**). Its subcellular localisation indicates that the gene encodes a protein called Heat shock protein 105 kDa, which is predominantly located in the cytoplasm and nucleus, and is also expressed in the extracellular matrix and cytoskeleton (**Figure 1B**). Gene-disease network interaction analysis revealed that HSPH1 is highly associated with a variety of cancers, including cutaneous melanoma, breast cancer, lung adenocarcinoma and squamous cell carcinoma of the head and neck, as well as inflammatory conditions such as pulmonary arterial hypertension and infections (**Figure 1C**). In order to gain insight into the correlation between HSPH1 gene expression and tumour diseases, we analysed the expression profiles of 33 tumours in the TCGA database. The results demonstrated that HSPH1 was significantly elevated in a range of tumours, including breast, ovarian, head and neck squamous cell carcinoma, and renal clear cell carcinoma. Furthermore, HSPH1 expression was also markedly elevated in NSCLC in comparison to normal tissues (**Figure 1D**).

### 3.2 Correlation between HSPH1 expression level and cancer stage in NSCLC tissues

**Figure 2:**
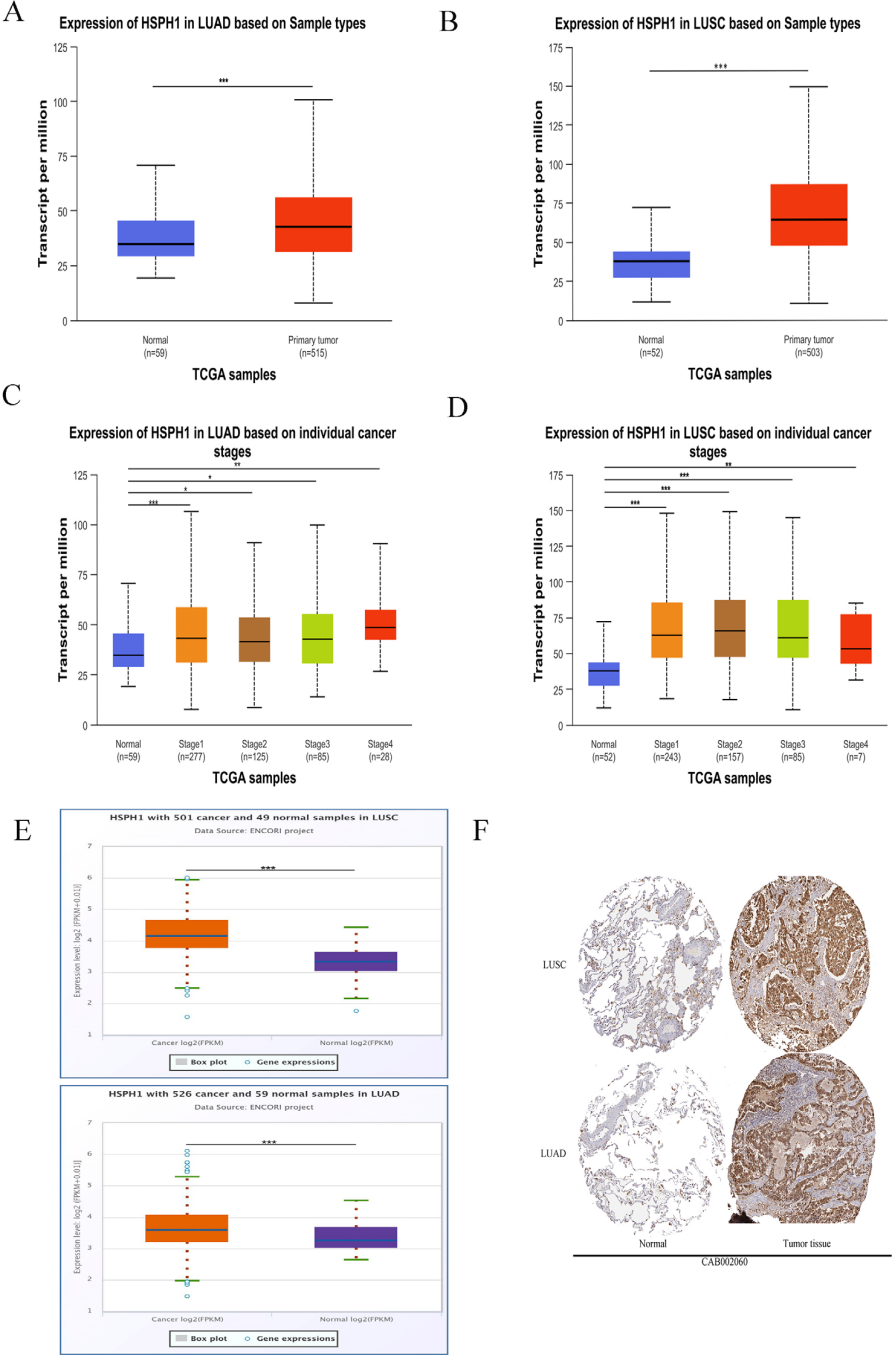
HSPH1 shows high expression in NSCLC and correlates with clinical stage. **(A, B)** UALCAN database analysis of HSPH1 expression differences in NSCLC tissues and normal tissues. **(C, D)** UALCAN database analysis of HSPH1 expression differences in different pathological stages of NSCLC. **(E)** Analysis of the expression difference of HSPH1 in NSCLC tissues and normal tissues based on ENCORI online database. **(F)** Immunohistochemistry (antibody no. CAB002060) results of HPA database showed the expression difference of HSPH1 at tissue protein level. (*P < 0.05, **P < 0.01, ***P < 0.001).

Based on the above HSPH1 expression differences in pan-cancer, we found that HSPH1 expression was significantly upregulated in NSCLC compared to normal tissues using UALCAN database analysis (**Figures 2A, B**). And according to the available histologic data from UALCAN, HSPH1 expression was significantly correlated with NSCLC stage (**Figures 2C, D**). It is worth mentioning that we also obtained the same results using ENCORI online database analysis (**Figure 2E**). In addition, to further explore whether there was a difference in HSPH1 protein expression in NSCLC tissues, we found that HSPH1 protein was significantly highly expressed in NSCLC tissues using immunohistochemistry results from the HPA database (**Figure 2F**).

### 3.3 Analysis of the effect of HSPH1 on the prognosis of NSCLC patients based on an online database

**Figure 3:**
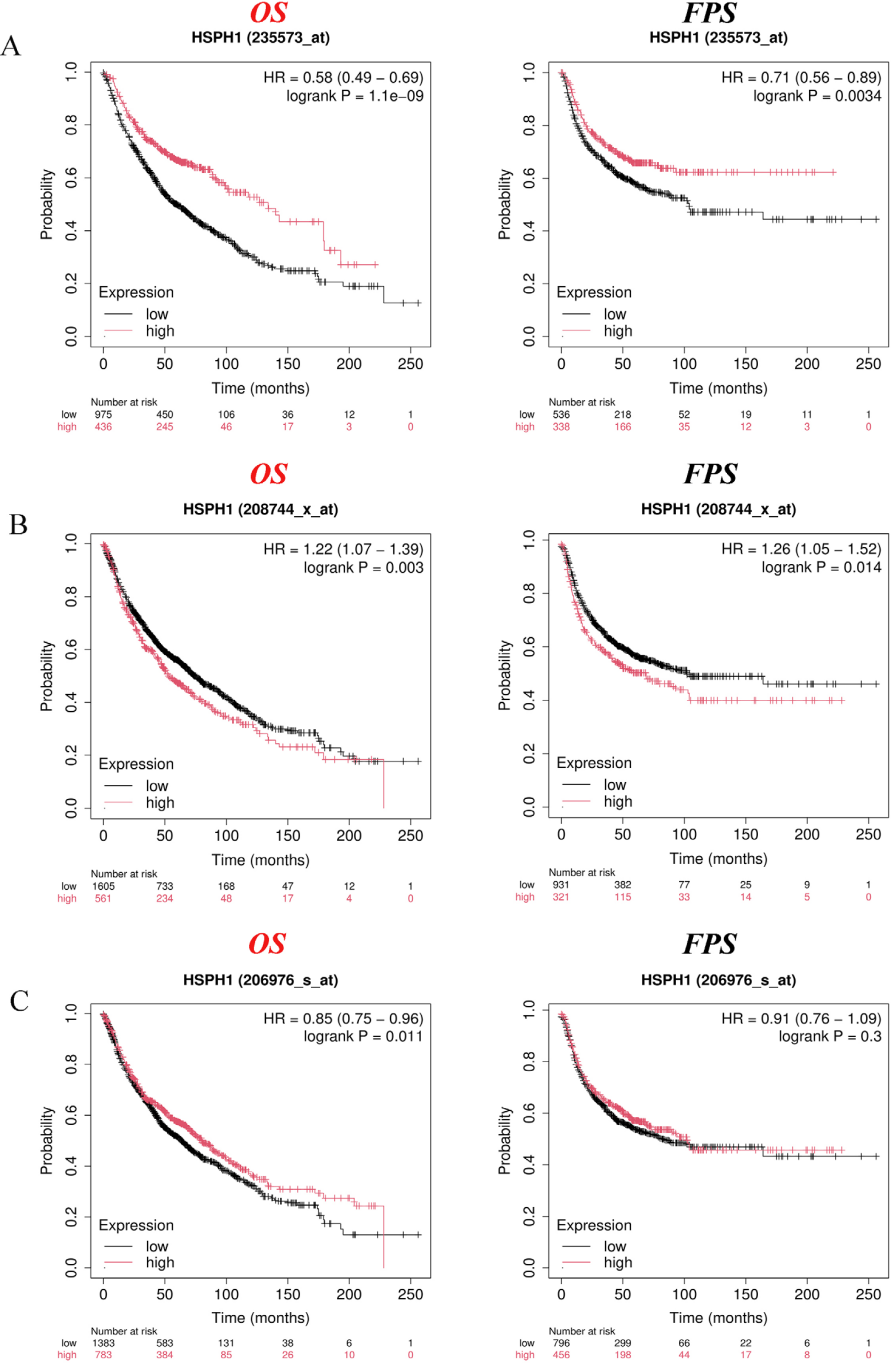
High HSPH1 expression is associated with poor prognosis in NSCLC patients. **(A)** KM database analysis of the relationship between HSPH1 expression and prognosis in NSCLC microarray data (235573_at). **(B)** KM database analysis of the relationship between HSPH1 expression and prognosis in NSCLC microarray data (208744_x_at). **(C)** KM database analysis of the relationship between HSPH1 expression and prognosis in NSCLC microarray data (206976_s_at).

To further evaluate the correlation between HSPH1 expression and the prognosis of NSCLC patients, we analysed three different microarray data (235573_at, 208744_x_at and 206976_s_at) using the KM database for the correlation between HSPH1 expression and NSCLC patients’ Overall Survival (OS) and First- progression Survival (FPS). Patient segmentation criteria are set by auto select best cutoff. The results demonstrated a significant correlation between HSPH1 expression and OS and FPS in NSCLC patients, as observed in both microarray data sets (235573_at and 208744_x_at) (**Figures 3A, B**). However, no correlation was identified between HSPH1 expression and FPS in microarray data set (206976_s_at) (**Figure 3C**). The aforementioned results indicated that elevated HSPH1 expression was linked to unfavourable patient outcomes. HSPH1 may therefore serve as a pivotal prognostic marker for NSCLC, particularly in the context of non-small cell lung cancer progression.

### 3.4 Interaction network of HSPH1

**Figure 4:**
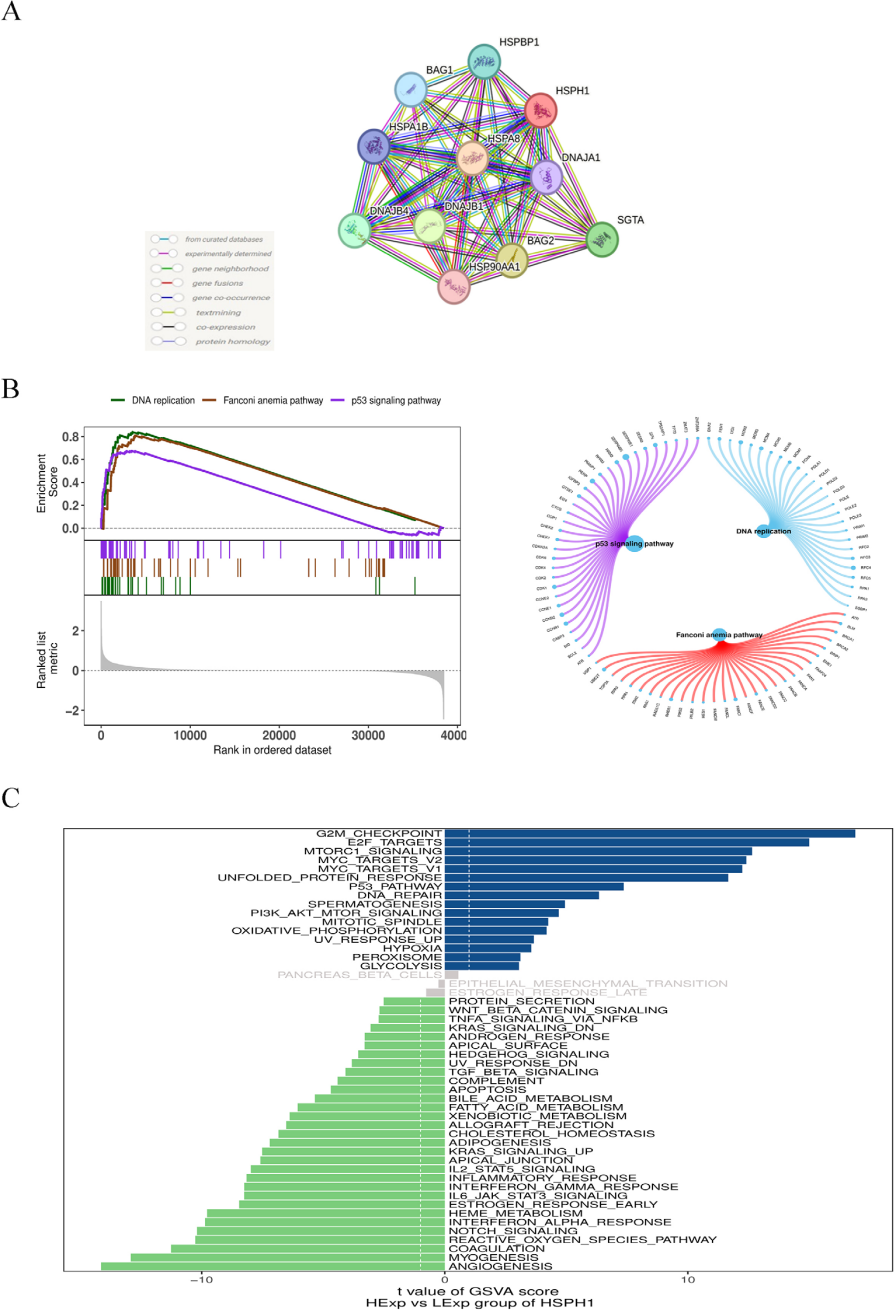
Single gene association and enrichment analysis of HSPH1 in NSCLC. **(A)** HSPH1 protein-protein interaction network analysis. **(B)** Gene set expression analysis. **(C)** Gene set variation analysis.

In order to elucidate the potential mechanisms by which HSPH1 affects NSCLC progression, we constructed the HSPH1 protein-protein interaction network (**Figure 4A**) through the STRING database, and obtained the 10 highest scoring predicted chaperone proteins as HSPA8(0.998), BAG2(0.995),DNAJB1(0.990),SGTA(0.987),DNAJB4(0.967),HSPBP1(0.963),BAG1(0.954),HSPA1B(0.953),DNAJA1(0.948),HSP90AA1(0.938). Then, we verified the relationship between the above proteins and OS in NSCLC patients through The Kaplan-Meier online database (**Supplementary Figure S1**), and found that the above proteins were significantly related to OS in NSCLC. Analysis through the GEPIA2 online database, we found that the expression of most interaction proteins was not significantly different in lung adenocarcinoma and lung squamous cell carcinoma, and the expression of DNAJB4 was different in LUAD, but not in LUSC (**Supplementary Figure S2**). The above evidence shows that HSPH1 may regulate stress adaptation in cancer cells through protein interaction networks, rather than relying on the expression of a single protein. The prognostic value of HSPH1 interaction protein was consistent in both subtypes, suggesting its potential as a pan-NSCLC biomarker, while DNAJB4 may be used as a co-marker of LUAD.

### 3.5 Pathway enrichment analysis of HSPH1 in NSCLC

In addition, we investigated the specific signalling pathways involved in HSPH1 to explore the potential molecular mechanisms by which HSPH1 affects disease progression. GSEA results showed that HSPH1 was enriched in signalling pathways such as p53 signalling pathway, DNA replication, and Fanconi anaemia pathway (**Figure 4B**). GSVA analysis showed that HSPH1 was positively correlated with signalling pathways such as G2M_checkpoint, E2F_targets, and mTORC1_signaling, and negatively correlated with pathways such as Angiogenesis, Myogenesis, and Coagulation (**Figure 4C**). These findings indicate that HSPH1 may play a role in the progression of NSCLC through a number of different pathways, including a potential pro-cancer role through the p53 and mTORC1 pathways.

### 3.6 Relevance of HSPH1 expression in immune infiltration

**Figure 5:**
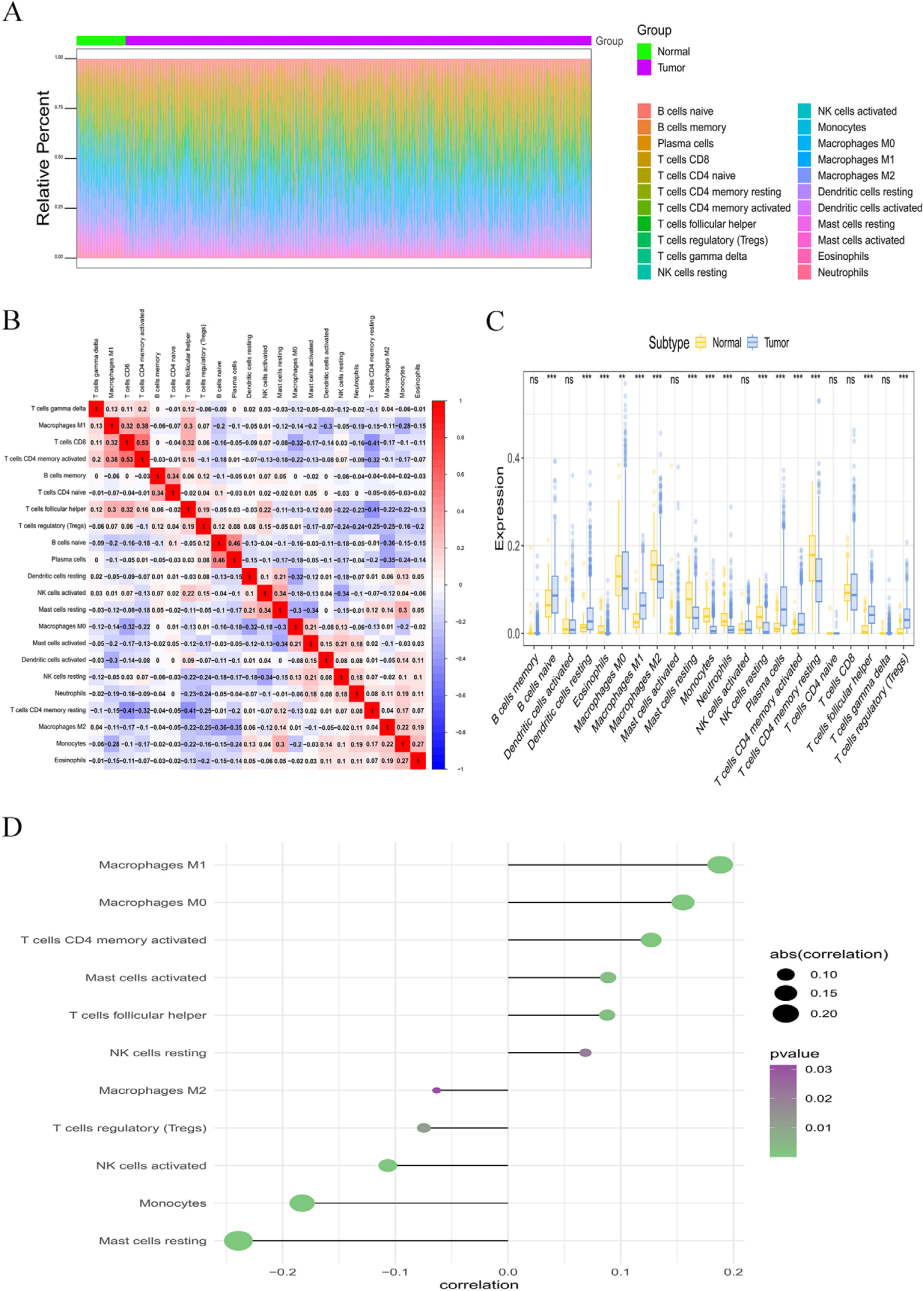
Correlation analysis between immune cell infiltration and HSPH1 in NSCLC. **(A, B)** Distribution of immune infiltration levels and immune cell correlations. **(C)** Intergroup comparison bar graphs detailing the differences in immune cell infiltration scores for the 22 immune cells mentioned above between HSPH1 high/low expression groups. **(D)** Lollipop plot of correlation between immune cell infiltration scores and HSPH1 gene expression in NSCLC patients. (**P < 0.01, ***P < 0.001, ns: no significance).

Subsequently, we investigated the correlation between HSPH1 gene expression and immune cell infiltration. The microenvironment is primarily constituted of fibroblasts, immune cells, extracellular matrix, multiple growth factors, inflammatory factors, and specific physicochemical features, among other elements. The microenvironment has a significant impact on the diagnosis of the disease, the survival outcome, and the sensitivity of clinical treatment. We showed the distribution of immune infiltration levels and immune cell correlations in different forms (**Figures 5A, B**). Compared with the control group, samples from the disease group had significantly higher levels of B cells naive, Dendritic cells resting, and Macrophages M1, and significantly lower levels of Macrophages M2, Mast cells resting, and T cells CD4 memory resting. (**Figure 5C**). We further explored the relationship between HSPH1 and immune cells and found that HSPH1 was significantly positively correlated with Macrophages M0, Macrophages M1, T cells CD4 memory activated, Mast cells activated, T cells follicular helper, etc. and significantly negatively correlated with Mast cells resting, Monocytes, T cell regulatory(Tregs),NK cells activated, Macrophages M2, etc. (**Figure 5D**). The above suggests that HSPH1 may promote disease progression by affecting the immune microenvironment.

### 3.7 Univariable and multivariable analysis based on TCGA database

**Figure 6:**
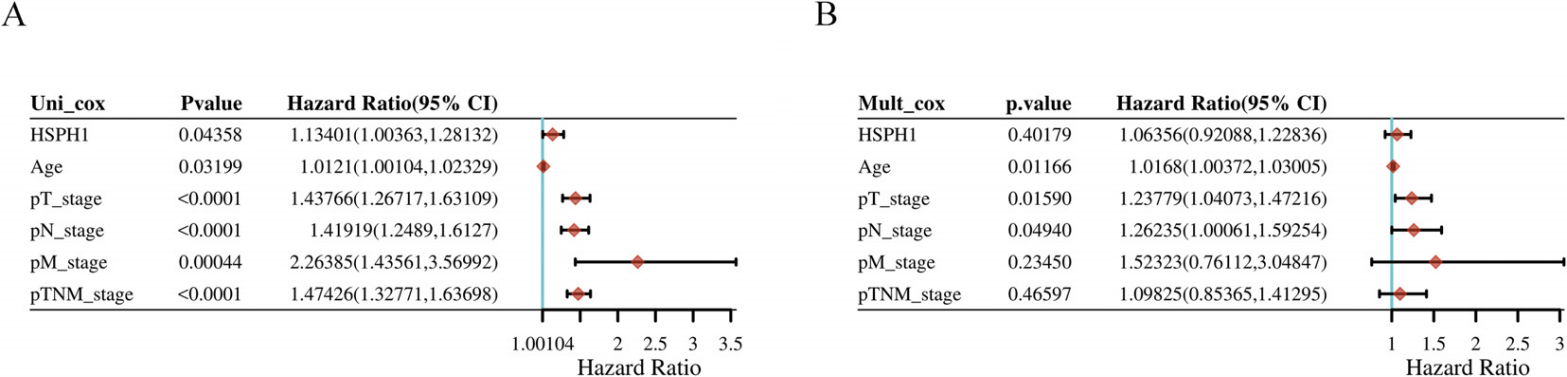
Univariable and multivariable analysis based on TCGA database. **(A)** Univariable analysis. **(B)** Multivariable analysis.

We constructed univariate and multivariate Cox regression models and generated forest plots based on the clinical data and HSPH1 expression in NSCLC-TCGA data. Multivariate Cox regression ended with overall survival (OS), and included variables including age, TNM stage, and HSPH1 expression. The results indicated that, in the univariate Cox regression analysis, the expression of HSPH1, age, and tumour stage were identified as risk factors (P < 0.05). In contrast, the multivariate Cox regression analysis revealed that only age and tumour T and N stage were risk factors (P < 0.05, **Figures 6A, B**). Accordingly, further validation would benefit from the inclusion of additional samples from our NSCLC cohort.

### 3.8 Evaluation of HSPH1 expression in lung cancer cell lines and NSCLC tissues

**Figure 7:**
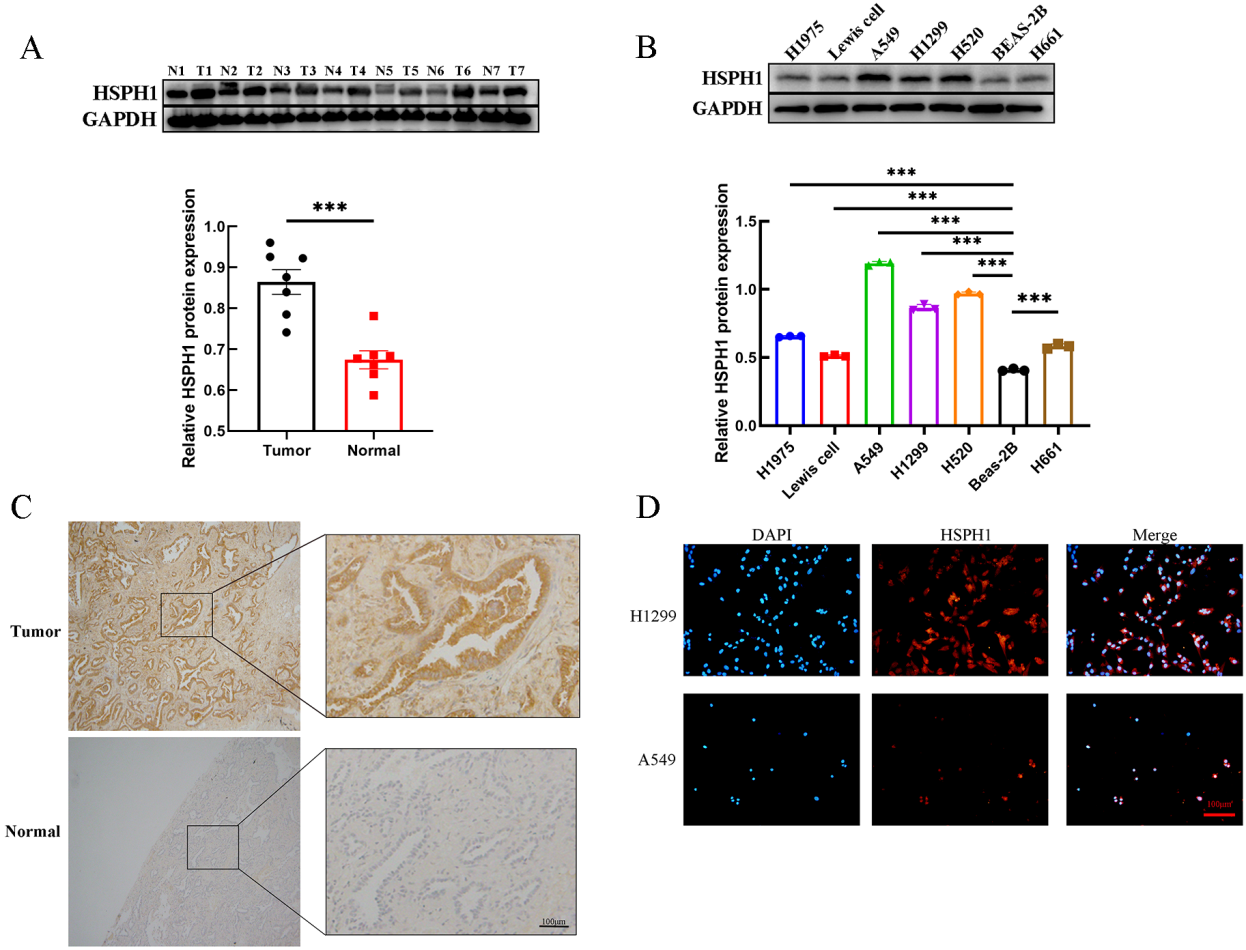
HSPH1 expression in NSCLC tissues and lung cancer cells. **(A)** Western-blot assay was used to determine the expression level of HSPH1 in 7 pairs of NSCLC tissues and paired normal tissues with gray value analysis, and the results were visualised using bar graphs. **(B)** Western-blot assay was used to determine the expression level of HSPH1 in several lung cancer cell lines with gray value analysis, and the results were visualised using bar graphs. **(C)** Immunohistochemical staining of tumour tissues and adjacent normal lung tissues from NSCLC patients. **(D)** The expression and distribution of HSPH1 in H1299 and A549 cells were determined by immunofluorescence. (T is NSCLC tumour tissue, N is adjacent normal lung tissue, ***P < 0.001).

To further validate the positive results of the bioinformatics analysis, we detected the expression of HSPH1 in human NSCLC tissues and lung cancer cell lines. The expression profile of HSPH1 in human NCSLC tissues detected by Western-blot revealed that in 7 pairs of fresh human NSCLC tissues, the expression of HSPH1 was significantly higher than that in normal lung tissues (**Figure 7A**). In addition, the expression level of HSPH1 in lung cancer cell lines (A549, H1299, H1734, H520, Lewis cell, Beas-2b, H1975) was also examined by Western-blot, and it was found that compared with normal lung epithelial cells Beas-2b, the expression of HSPH1 was significantly higher in the remaining lung cancer cells, and the particular the difference was more obvious in both A549 and H520 cells (**Figure 7B**). Importantly, immunohistochemical staining also revealed higher HSPH1 expression in NSCLC tissues than in normal lung tissues (**Figure 7C**). In addition, we used cellular immunofluorescence to detect the expression distribution of HSPH1 in two NSCLC cells, H1299 and A549, and found that HSPH1 is widely expressed in the nucleus and cytoplasm, which is consistent with the results of the above analysis (**Figure 7D**). Overall, the above experimental results confirmed that the expression of HSPH1 in human LUAD tissue was higher than that in normal tissue, which was consistent with the results of bioinformatics analysis.

### 3.9 Correlation analysis between HSPH1 expression and the clinicopathological parameters of NSCLC

The expression grade of HSPH1 was used to categorise all NSCLC patients into high and low expression groups in order to explore the correlation between the expression level of HSPH1 and the clinicopathological parameters of NSCLC (**Table 1**). The results indicated that the expression level of HSPH1 in NSCLC was significantly correlated with T (representing the size of the tumour and the extent of the primary tumour), N (representing lymph node involvement), M (referring to distant metastasis), and histology stage and clinical stage (P < 0.05). However, no statistically significant correlation was observed between the expression level of HSPH1 in NSCLC and age, gender, or smoking history (P > 0.05).

**Table 1:** Correlation between HSPH1 expression profile and clinicopathologic features of NSCLC.

Clinicopathological Features	n	HSPH1		*P* value^#^
		High expression (n=50)	Low expression (n=45)	
Gender				
Male Female	5342	30 20	23 22	0.384
Age (years)				
≥60 <60	6332	34 16	29 16	0.714
Smoking index				
≥nde <400	34 61	19 31	15 30	0.636
T stage				
T1/T2 T3/T4	4055	13 37	27 18	0.001*
N stage				
N0 N1/N2/N3	6134	23 27	38 7	<0.001**
M stage				
M0 M1	78 17	37 13	41 4	0.030*
Clinical stage				
I/II III/IV	5045	15 35	35 10	<0.001**
Histology stage				
Well Poorly	64 31	26 24	38 7	0.001*

^#^Chi-square test,*P<0.05, **P<0.01.

### 3.10 Prognostic significance of HSPH1 expression in NSCLC

**Figure 8:**
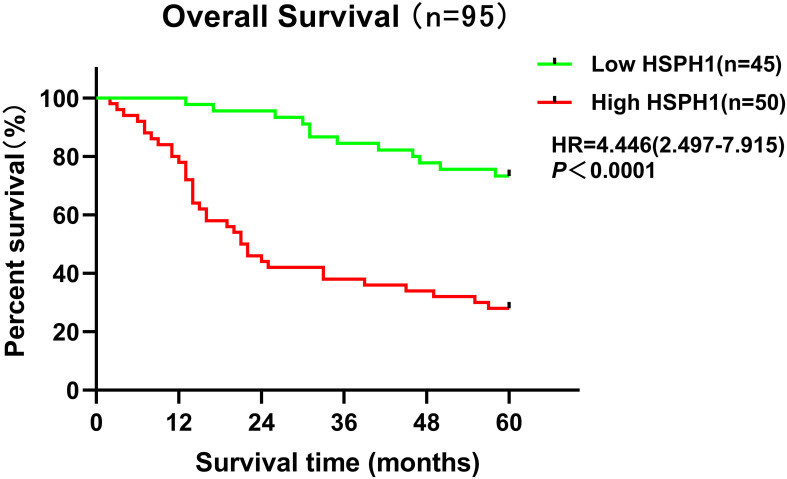
Kaplan-Meier survival analysis of HSPH1 expression levels and NSCLC patients based on clinical data.

The Univariate Cox regression analysis demonstrated (**Table 2**) that HSPH1 expression level, TNM, Clinical stage and Histology stage could be used as prognostic factors of NSCLC (P < 0.001). And multivariate Cox regression analysis further revealed (**Table 2**) that HSPH1 expression level, distant metastasis (M), Clinical stage and Histology stage were independent prognostic factors for NSCLC. Most importantly, HSPH1 expression was significantly associated with OS in NSCLC patients on the Kaplan-Meier survival curve, and high HSPH1 expression was significantly associated with shorter OS (**Figure 8**). Therefore, the above results suggest that HSPH1 can be used as an independent prognostic factor in NSCLC patients, and its high expression often represents a poor prognosis for patients, so HSPH1 has great potential to become a prognostic marker for NSCLC.

**Table 2:** Univariate and multivariate analysis of prognostic factors for 5-year overall survival in NSCLC.

Characteristic	Univariate analysis			Multivariate analysis		
	HR	95%CI	P	HR	95%CI	P
HSPH1 expression						
High vs Low	4.586	2.373~8.860	<0.001**	2.515	1.205~5.247	0.014*
Gender						
Male vs Female	1.710	0.946~3.090	0.076			
Age (years)						
≥60 vs <60	1.244	0.675~2.292	0.484			
Smoking index						
≥400 vs <400	1.212	0.679~2.161	0.515			
T stage						
T3~4 vs T1~2	5.546	2.583~11.908	<0.001**			
N stage						
N1~3 vs N0	9.567	4.990~18.345	<0.001**			
M stage						
M1vs M0	8.681	4.384~17.193	<0.001**	4.892	2.345~10.205	<0.001**
Clinical stage						
III/IV vs I/II	13.200	6.053~28.782	<0.001**	3.971	1.370~11.511	0.011*
Histology stage						
Poorly vs Well	6.289	3.414~11.585	<0.001**	2.401	1.223~4.711	0.011*

HR, hazard ratio; CI, confidence interval; *P<0.05, **P<0.01.

## 4 Discussion

As the global incidence and mortality of non-small cell lung cancer (NSCLC) continues to rise, its treatment remains a major challenge (22). Despite advances in available therapies (e.g., targeted and immunotherapies), the prognosis for patients with advanced disease is generally poor, with a 5-year survival rate of 26.5% (23). Notably, about 60% of patients with advanced disease carry specific molecular variants that may benefit from precision-targeted therapy (24). Therefore, there is an urgent need to develop novel biomarkers for early diagnosis and prognostic assessment.HSPH1, as a molecular chaperone, plays a key role in cellular stress response and protein homeostasis. In this study, for the first time, we systematically revealed the high expression characteristics of HSPH1 in NSCLC and its value as a potential biomarker by multidimensional analysis.

First, we analysed the characterisation of HSPH1, including its chromosomal and subcellular localisation, using online databases and found that HSPH1 was highly associated with a variety of cancers through gene-disease network interaction analysis. A pan-cancer analysis based on the TIMER database also confirmed that HSPH1 expression was significantly elevated in several cancers.

Second, in this study, HSPH1 was found to be significantly highly expressed in NSCLC tissues, and its expression level was strongly correlated with tumour stage (T, N, M) and clinical stage. Multivariate Cox regression analysis further confirmed that HSPH1 was an independent prognostic factor in NSCLC, and its high expression was significantly associated with advanced stage (T/N/M) and poor histological differentiation. Partial contradiction of this finding with the TCGA data (e.g. HSPH1 was not an independent factor in the multivariate analysis) may be due to the small clinical sample size (n=95) and single-centre bias in this study, which needs to be validated by a multicentre large sample cohort in the future. These results are consistent with the pro-oncogenic role of HSPH1 in pan-cancers such as breast cancer and oral squamous cell carcinoma, where high expression of HSPH1 is also associated with poor prognosis (9, 25). In particular, we have demonstrated the high expression of HSPH1 in clinical samples by immunohistochemistry and Western blot experiments, further supporting its reliability as a biomarker for NSCLC.

Protein interaction network analysis revealed that HSPH1 forms a complex with chaperone proteins such as HSPA8, BAG2, and DNAJB1, which may promote tumour progression by regulating stress response and cell survival (26).Although the expression of these proteins does not differ significantly between lung adenocarcinoma (LUAD) and lung squamous cell carcinoma (LUSC), the concordance in their prognostic value suggests that HSPH1 may regulate stress adaptation through dynamic changes in post-translational modifications (e.g., phosphorylation, acetylation) or subcellular localisation. For example, the active conformation of HSP90 is dependent on binding to client proteins such as AKT (27). HSPH1 may enhance its function through a similar allosteric mechanism, but the specific target remains to be experimentally verified. The specific high expression of DNAJB4 in LUAD further supports its potential as a subtype marker, and its spatial and temporal distribution in the tumour microenvironment can be resolved in the future using single-cell spatial transcriptomic techniques.

GSEA analysis reveals that HSPH1 is enriched in the p53 signalling pathway, DNA replication and Fanconi anaemia signalling pathway, that p53 pathway inactivation is a common event in NSCLC, and that TP53 mutations are widespread in most human cancers (28). HSPH1 inhibits DNA repair by negatively regulating the p53 signalling pathway, exacerbates genomic instability and may promote cancer by inducing DNA replication stress (29). In addition, GSVA analysis showed that HSPH1 was significantly associated with the G2/M checkpoint, E2F targets and the rapamycin complex 1 (mTORC1) pathway, suggesting that it drives tumour proliferation by interfering with cell cycle regulation (e.g. inhibiting DNA damage repair) and metabolic reprogramming (e.g. activating mTORC1). However, whether HSPH1 is involved in promoting tumour development through the above-mentioned pathways requires further functional experiments.

Tumour-associated immunosuppression is a major cause of treatment failure in NSCLC. In this study, we found that HSPH1 is positively correlated with M1 macrophage infiltration, but can also promote immune escape by inducing M1 to M2 polarisation. This phenomenon is distinct from the mechanism by which HSP70 directly activates M2 macrophages via TLR4 (30). The functional heterogeneity of HSP family members in immune regulation was highlighted. In addition, HSPH1 was negatively correlated with inhibition of NK cell activity and regulatory T cells (Tregs), suggesting that it may promote tumour immune escape by modulating the immunosuppressive microenvironment (31). In particular, the positive correlation between HSPH1 and activated mast cells suggests that it may promote angiogenesis and metastasis by releasing mediators such as histamine and VEGF (32). This provides a rationale for co-targeting HSPH1 and anti-angiogenic drugs such as bevacizumab.

The results of this study are highly consistent with previous reports on the function of HSPH1 in solid tumours. For example, high expression of HSPH1 in hepatocellular carcinoma is associated with tumour metastasis (33). In colorectal cancer, HSPH1 promotes tumour progression by activating STAT3 (34). However, the present study systematically analysed the expression pattern, prognostic value and immune relevance of HSPH1 in NSCLC for the first time, providing new evidence for its clinical application. The high expression of HSPH1 was significantly associated with poor prognosis of NSCLC patients, suggesting that it may be a potential therapeutic target. In the future, it is necessary to further validate the mechanism of HSPH1 in NSCLC through functional experiments and explore its correlation with the efficacy of immune checkpoint inhibitors. In addition, the construction of HSPH1-related prognostic models using multi-omics data may optimise the individualised treatment strategy for NSCLC patients.

## 5 Conclusions

In conclusion, the findings of this study demonstrate that HSPH1 is markedly expressed in NSCLC and plays a pivotal role in NSCLC pathogenesis and progression. Furthermore, its elevated expression is significantly associated with a poor prognosis in NSCLC patients. Consequently, HSPH1 may emerge as a potential biomarker and therapeutic target for the diagnosis of NSCLC. A more detailed examination of HSPH1 in NSCLC will assist in determining the precise mechanism through which HSPH1 contributes to cancer development, thereby enhancing the potential for utilising HSPH1 in clinical settings.

## Data Availability

The original contributions presented in the study are included in the article/**Supplementary Material**. Further inquiries can be directed to the corresponding authors.
