# Supplementary material for: Correlation of heat shock protein 1 expression with progression and prognosis of non-small cell lung cancer
Source: Front Oncol. 2025 May 6;15:1553248. doi: 10.3389/fonc.2025.1553248 (PMC12088961; doi:10.3389/fonc.2025.1553248)
Supplement: Supplementary file 1 [file DataSheet1.pdf]

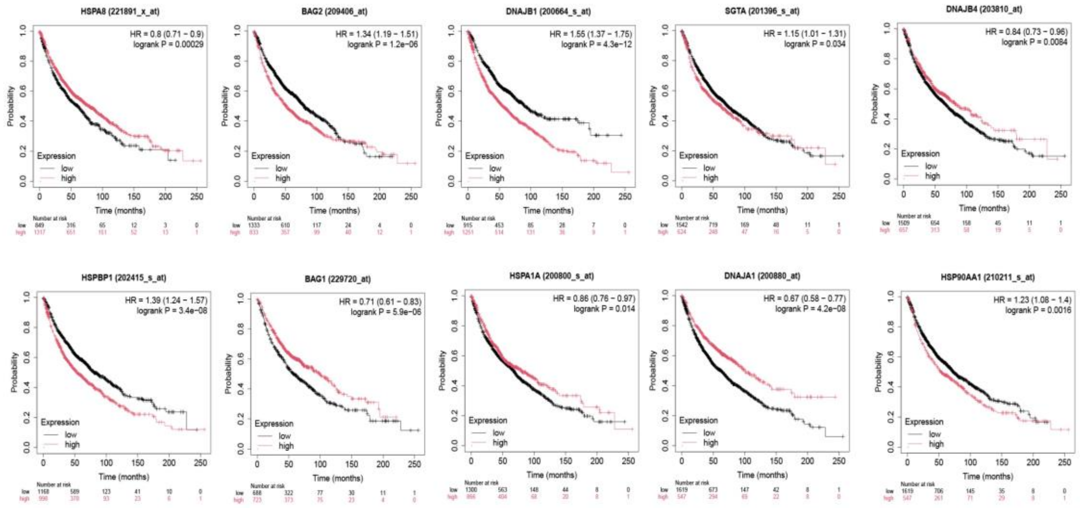

Supplementary Figure 1: The Kaplan-Meier online database investigated the relationship between HSPH1-interacting proteins and OS in patients with NSCLC.

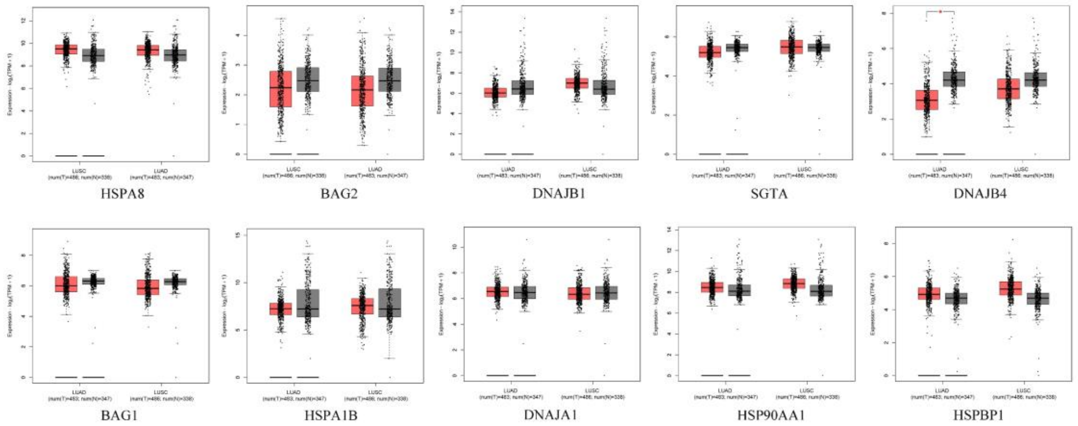

Supplementary Figure 2: Differences in expression of HSPH1 interacting protein in lung adenocarcinoma and lung squamous cell carcinoma were analyzed based on GEPIA2 database.
